# Full exclusion during COVID-19: Saudi Deaf education is an example

**DOI:** 10.1016/j.heliyon.2021.e06536

**Published:** 2021-03-15

**Authors:** Abdullah Madhesh

**Affiliations:** Department of Special Education, Shaqra University, Shaqra, Saudi Arabia

**Keywords:** Inclusion, Exclusion, Deaf students, COVID-19

## Abstract

The ongoing COVID-19 pandemic has changed the world in many aspects, including educational provision. Most countries around the globe suspended schools and provided alternative educational methods. And Saudi Arabia is not an exception. This study aims to investigate the situation of Saudi Deaf^1^ students in compulsory education during the suspension of schools. The study presents a discussion on the alternative education methods and content provided by the Ministry of Education in Saudi Arabia during this crisis and is followed by individual semi-structured interviews with eighteen teachers for Deaf students. Three categories were formulated: (1) Deaf students' educational status during the suspension of schools (full exclusion); (2) teachers' responses to the exclusion of their students; and (3) reasons for the full exclusion. The findings suggest that governments and educational organisations in Saudi Arabia need to facilitate inclusion practices for Deaf students in alternative educational methods and tools, including distance education.

## 1 Introduction

The COVID-19 outbreak brought about several changes in countries around the world and affected many aspects of life. The fast spread and infection rate of the virus forced most countries to limit or prohibit many activities that have large gatherings of people. The pandemic affected the movements of trade both locally and globally (Baldwin and Tomiura 2020). Several countries approved quarantine measures and restricted the movement of citizens (Kraemer et al., 2020). Even travelling was completely suspended in many countries (Chinazzi et al., 2020). Many people experienced different psychological problems as a consequence (Wang et al., 2020).

Indeed, the education system was one of the first institutions affected by COVID-19. Almost all infected countries shut down all educational organisations and asked all stakeholders to stay home. According to Zhou et al. (2020), many schools around the world were closed and directed to use alternative educational methods to continue teaching their students.

### 1.1 Alternative educational methods during COVID-19

The pandemic forced many schools around the globe to consider alternative educational methods and strategies to meet the educational needs of affected students. The entire education system in many countries was changed and shifted to be more learner-centred than teacher-centred. Indeed, flexible learning (Gordon 2014), in this pandemic, compensated for students' lack of physical attendance at school. The concept of flexible learning depends on new and alternative strategies for delivering education contents that rely on technological developments such as communication tools and electronic platforms (Ryan and Tilbury 2013). E-learning (technology-enhanced learning) is an example of a flexible learning strategy (Cook et al. 2019). However, in this case, e-learning (online learning) was used as a tool to enhance distance education for affected students.

Distance education utilises telecommunication technologies to provide different educational materials and curriculums for students without physical attendance by employing different platforms, websites and applications (Simonson et al. 2019). Therefore, in this pandemic, distance education replaced traditional learning where many students were prevented from attending schools and asked to complete their education online from their homes. For example, in China, the Ministry of Education closed all the schools nationwide and established an alternative program called ‘School's Out, But Class's On’ to provide distance educational services for all students (Zhou et al., 2020, 502). This program according to Zhou et al. (2020) included using internet platforms and TV channels to enhance home study during the crisis.

In the USA, all schools around the country were closed. The USA quickly adopted a distance education system that included video-conference technologies, different websites, electronic platforms, applications and/or various databases. Most schools established innovative virtual learning forms to serve American students and teachers during the COVID-19 pandemic (U.S. Department of Education 2020).

In Saudi Arabia, all educational organisations were shut down on March 9, 2020 (Saudi Press Agency 2020). All students, teachers and administrators were asked to stay home and shift to distance education services. According to UNESCO (2020), the Ministry of Education in Saudi Arabia provided some alternative methods to support remote education during the crisis, such as 20 satellite TV channels called IEN TV Channels to provide live broadcasting of lessons and educational activities for all school levels (UNESCO 2020). An interactive platform called Future Gate was used to provide virtual classrooms and some digital content (UNESCO 2020). An official online learning portal called Ien National E-portal was utilised to provide some course materials for pre-primary up to secondary education. An E-learning platform called Vschool as well as many resources by grade level and subject area were also used (UNESCO 2020). Vschool is a platform that was created by the Ministry of Education and includes all the above methods (IEN TV Channels, Future Gate and Ien National E-portal) on one platform.

Many other countries also closed down their schools and transferred to alternative remote methods. However, transferring from physical attendance education to distance learning faced and is still facing some difficulties and barriers. First, is the availability of required technologies for this format of education, such as computers, tablets and smartphones. Some students may not have the essential technologies for distance education. Second, the lack of an internet connection or the internet speed for remote learning is an obstacle for many students (Xu and Xu 2019). In the USA, for example, more than 17% of American students do not have computers at home and about 18% do not have access to home broadband internet (Melia et al. 2019). Additionally, some students live in areas where the internet network connection is poor. Third, the nature of course or content could constitute a barrier to applying online learning (Simonson et al. 2019). For instance, some subjects require practical experiences that need to be physically done by students. Additionally, the required skills of using some technologies and materials in distance education may create a struggle for some students (Xu and Xu 2019). Finally, the nature of the learner could pose a difficulty for distance education, e.g. a student with a disability. According to Chadwick and Wesson (2016, 14), ‘the interfaces of ICT and associated programmes and applications can be off-putting, challenging and extremely difficult to use for those with sensory, cognitive, and physical disabilities’.

### 1.2 Distance education for Deaf students

Deaf students face not only the above-discussed difficulties and problems related to distance education but also some additional challenges. While there is an increase in online learning for Deaf learners, this tendency has not always been easy and faces some potential barriers. For instance, McKeown and McKeown (2019) confirm that for Deaf students, some barriers exist at many levels of distance learning such as the structural nature of most online courses in terms of content, tools, presentation methods, and communication requirements. Moreover, Deaf students, most times, are excluded from distance education as a result of the preconception that they need more specialised educational approaches (Liasidou and Symeou, 2018). Indeed, it is no surprise that the extent of exclusion increased during COVID-19 (Mantzikos and Lappa, 2020).

### 1.3 Education of students with disabilities during COVID-19

Schooling services for students with and without disabilities were suspended as a response to the COVID-19 pandemic in many countries around the world. This was done to protect students from contracting the virus and possibly spreading it to family members. One unexpected outcome of the pandemic is that it highlighted the barriers that students with disabilities face in the classroom. Even before this pandemic, students with disabilities are at greater risk for exclusion from accessing essential services (Bernard et al., 2020) including proper education compared to their peers without disabilities (Gilmour et al. 2019). Indeed, these students are predominantly left behind during disasters, crises and emergencies such as in the case of COVID-19 (Kuper et al., 2020). However, some efforts were made to support educational services for students with disabilities during this crisis.

In the USA, for instance, the U.S. Department of Education (2020) confirmed that individualised remote education must continue during the COVID-19 pandemic for all students around the country, including students with disabilities. Additionally, Q&A guidance was released by the U.S. Department of Education to provide instructions and recommendations to service students with disabilities during the COVID-19 outbreak (The National Conference of State Legislatures, 2020). In the UK, the Department for Education developed a list of resources, such as the Brain Parade App, HelpKidzLearn Website, Sensory App House Ltd and Visuals2Go App (with a focus on accessibility and inclusivity) as alternative tools for students with disabilities to engage in distance education during the crisis (Department for Education, UK 2020). However, in Saudi Arabia, from a three–week observation of the Ministry of Education website and its news and all released alternative distance learning tools, it was realised that students with disabilities seemed to be excluded. As a result, this study was conducted to investigate the situation of Deaf students in Saudi Arabia and to open the door for more studies to examine the status of other students with different types of disabilities.

## 2 Methodology

To achieve the aim of this study, first, observation of the 20 Ministry of Education educational channels that were used during COVID-19 to provide educational lessons for students at all levels was conducted. Second, the contents of the Ministry of Education Website and other methods used in distance education were analysed. Third, individual semi-structured interviews were conducted with eighteen teachers that teach Deaf students in all education levels of compulsory education (six teachers from every level). Compulsory education in Saudi Arabia includes three levels: primary school, intermediate school (middle–school) and high school.

### 2.1 Observation

Although observation is a technique commonly used in behavioural studies, the researcher chose to use it as it was appropriate to the study. Following in the footsteps of Bakeyeva et al. (2020); Özkanal et al. (2020) observation of the IEN TV channels began when the Saudi Ministry of Education announced the suspension of attending schools for all students around the country, as a response to the COVID-19 outbreak and approved distance learning as an alternative educational strategy. The 20 TV channels belong to the Saudi Ministry of Education and were employed to provide curriculum lessons in all education levels of compulsory education. Three weeks, five weekdays per week, six hours every day, from Sunday 29 March 2020 to 16 April 2020, were spent observing these channels to examine their educational services for students with disabilities in general and Deaf students specifically. The researcher carefully observed these 20 TV channels and recorded any teaching materials or practices that were aimed at Deaf students. Hand-written notes were taken during the observation.

### 2.2 Analysing the provided contents

An analysis of the contents from the Saudi Ministry of Education as a response to schooling suspension resulting from the COVID-19 outbreak was performed. Analysing procedures covered Future Gate and the Ien National E-portal. In addition to these platforms, 28 news articles starting from March 11, 2020, two days after the suspension of schools until June 14 that were published on the Ministry of Education Website were also carefully analysed. The researcher carefully examined all the gathered content and noted any teaching materials or practices that were aimed at Deaf students. Hand-written notes were taken during the analysis.

### 2.3 Interviews

#### 2.3.1 Ethical consideration

The head of research––rather than the entire committee––from the Shaqra University Ethics Committee (SUEC) ethically approved this study, since it was categorised as low–risk (i.e. unlikely that any harm would come to participants as a result of participating in the study). The teachers were invited to participate in the interviews by sending a message to WhatsApp groups. The message described the purpose of the study and the nature of participating. After receiving an agreement from all teachers, they were asked to sign an informed consent form before the interviews. The informed consent included the description of the study and pointed out that participants could withdraw from the study at any time without any consequence. Participants were guaranteed confidentiality and the use of pseudonyms instead of their real names.

#### 2.3.2 Subjects

A purposeful sampling (Shaheen et al. 2019) method was applied to ensure that there was an equal number from every level of compulsory education and gender. A total of eighteen teachers of Deaf students participated in this study (six teachers from every level; three female and three male). All teachers teach in so-called self-continued classrooms for Deaf students, alternatively referred to as isolated classrooms (see Madhesh, 2019). Table 1 provides detailed information about the participants.

**Table 1:** Participant details.

Pseudonym	Age	Gender	School level
Sami	34	Male	Primary school
Asma	33	Female	Primary school
Nasser	28	Male	Primary school
Fatima	26	Female	Primary school
Jamal	45	Male	Primary school
Slma	41	Female	Primary school
Ahmed	38	Male	Intermediate school
Sarah	28	Female	Intermediate school
Ryan	26	Male	Intermediate school
Rana	33	Female	Intermediate school
Faisal	42	Male	Intermediate school
Abeer	45	Female	Intermediate school
Khaled	27	Male	High school
Wafa	36	Female	High school
Fahad	35	Male	High school
Aleen	26	Female	High school
Omer	28	Male	High school
Aamaal	42	Female	High school

#### 2.3.3 Procedure and instrument

The interviews aimed to elicit information regarding the education of Deaf students' education during the COVID-19 pandemic. First, the participants were asked about what happened to these students after schools were suspended. Additionally, all related matters to this case were discussed with the participants, such as the educational effects, the difficulties associated with the alternative methods, and who was responsible for the education of Deaf students during the crisis.

All interviews were conducted via phone calls. They were audio-recorded and transcribed. The transcripts were analysed qualitatively using a thematic analysis method (Terry et al., 2017). In the first round of analysis, the situations of Deaf students after the suspension of schools that the participants mentioned during the interviews were examined. In the second round, the focus lay on the effects of the suspension––what happened to their Deaf students, reasons, difficulties and responsibilities around this issue.

From the above analysing procedures, the findings were structured into three main categories. These categories were: (1) Deaf students' educational status during the suspension of schools (full exclusion); (2) teachers' responses to full exclusion of their students; and (3) reasons for full exclusion.

## 3 Findings

All analysing procedures for the provided contents and gathered data from the interviews were theoretically based on the Inclusive Education Theory (Slee, 2011, Slee, 2018, 2019). This theory indicates that many exclusionary practices are entrenched in many schools, and students with disabilities are treated as an additional population most of the time. From these analysing procedures, three major categories were structured from observing the above–mentioned contents and analysing gathered data from all eighteen interviews. These categories will be presented separately in the following sections.

### 3.1 Deaf students' educational status during the suspension of schools (full exclusion)

#### 3.1.1 Observation of TV channels

Surprisingly, all the contents of these channels were organised for students without disabilities and nothing was aimed at students with disabilities, especially Deaf students. There were no closed captions in any of the channels, nor were there on–screen Deaf interpreters. The three-week observation revealed that the educational programs were designed for hearing students without any consideration given to the needs of Deaf students.

#### 3.1.2 Analysis of educational content

From the analysis for the provided contents during the suspension of schools that resulted from COVID-19, it was concluded that there were no alternative methods dedicated specially for Deaf students to provide them educational services during the pandemic. First, Future Gate is an electronic gate that was created by the cooperation between the Saudi Ministry of Education and Tatweer Education Holding Company. This gate provides a set of educational services that aim to improve the skills of many beneficiaries including the Ministry and schools' administrators, teachers and students. From analysing the introductory material of this gate and contents, there was nothing for students with disabilities in general and Deaf students especially. Indeed, they were not mentioned at all.

Second, the Ien National E-portal is an electronic portal that was created by the Ministry of Education. It includes all national curricula for all education levels of compulsory education. From analysing this E-portal, it covered the national curricula for two categories of students with disabilities, namely students with intellectual disability and Deaf students. However, for students with intellectual disability, the curricula were transferred from textbooks to PDF forms without any enrichment or supporting contents as with the content for students without disabilities. With the content for Deaf students, the national curricula were uploaded with some enriching content, but they were not dedicated to Deaf students as all materials were free from sign language.

Finally, an analysis of all the news related to COVID-19 that was published on the Ministry of Education Website was conducted. There were 28 news articles starting from March 11, 2020, two days after the suspension of schools until June 14. There was an absence of mentioning students with disabilities in all 28 published articles. It was concluded that no one method considers the needs of Deaf students. For example, all electronic and online platforms have no tools, including sign language as the main and only communication tool for all Deaf students around the country. As a consequence of these outcomes, it was determined that an in-depth investigation was required. Thus, interviews with teachers of Deaf students were carried out.

#### 3.1.3 Interviews

All the participants confirmed that the academic year ended for all Deaf students when school was suspended in Saudi Arabia. Additionally, all eighteen participants agreed that there were no alternative educational methods dedicated to Deaf students during the crisis. A quotation from Sami who is a teacher for Deaf students in primary school confirmed this:

The school year of these students [Deaf students] was over once the suspension of study was implemented. All students were asked to stay at home and no follow-up instructions or any educational services for them were provided since that time (Sami).

Sarah, who is a middle–school teacher for Deaf students, reported the same situation for her Deaf students after the suspension of school:

Once the Ministry of Education announced that schools were to be shut down, our Deaf students stayed at home and did nothing, they do not have any alternative tools to complete their studying and this year is finished for them (Sarah).

Wafa who is a high school teacher for Deaf students stated the same case with her students:

Nothing happened for them [her Deaf students]. When the suspension of schools started, they were directed to stay at home and their studying was over at this point. Although the Ministry of Education offered some educational means for general students [students without disabilities] such as the IEN TV Channels, there is nothing for Deaf students. All these channels were designed for regular students, not one channel had a sign language translation service (Wafa).

The above quotes explain how Deaf students in Saudi Arabia had no option to complete their studying during this pandemic. Indeed, all the participants provided similar statements which were shortened to avoid repetition.

Most participants (sixteen from eighteen) mentioned how this crisis increased the exclusion of Deaf students. Though all participants confirmed that the school services ended as a result of COVID-19, 16 participants confirmed that the exclusionary practices increased during the pandemic.

Nasser, for instance, who is a primary school teacher, stated that:

Even though Deaf students suffer from exclusionary practices before COVID-19, this pandemic contributed to increasing this exclusion. They now are fully excluded at home with nothing to do (Nasser).

Rana, a middle–school teacher for Deaf students, also confirmed this viewpoint:

My students are now facing greater exclusion as a result of this pandemic. Although they were previously excluded partially in self-contained classrooms, they were in a much better situation than now (Rana).

Additionally, Khaled, who is a high school teacher for Deaf students stated that:

The absence of any form of educational services constituted a big change in the daily lives of these students. They are isolated and excluded in their homes. Most families do not communicate very well with these students as a result of the absence of sign language in many homes. In addition, most TV channels do not provide translation services for their contents, which leaves the Deaf student in an isolated world (Khaled).

These quotes present how the exclusionary practices against Deaf students in Saudi Arabia increased during COVID-19.

### 3.2 Teachers' responses to the full exclusion of their Deaf students

The participants were asked about their efforts as a response to the exclusion of their students from educational services during the suspension of schools. Only one teacher from eighteen tried to provide some compensatory efforts, but he found some barriers. He mentioned his experience in the following way:

After our school closed, I communicated with nine of my students and created a WhatsApp group for them to provide some lessons during this time. But this action failed because only four students had internet services. Also, only two of these four sometimes responded to my messages and questions (Fahad).

The above quote shows how the teacher tried to help his students during this pandemic, but he failed to provide educational services during this time.

The other seventeen teachers did nothing during this crisis. As their responses were the same, only some have been provided to avoid repetition. Asma who is a primary school teacher for Deaf students confirmed that:

Honestly, I did nothing, indeed, I couldn't. All students stayed home immediately, and we did not have a plan B to help these students out of school. It is difficult to keep in touch with them (Asma).

### 3.3 Another example

I couldn't complete this year's study plan with my students. We were asked to give them the same grades that they achieved in the first semester. Indeed, I did not have any way to complete this semester, and no one gave us any alternative method or tools to teach these students during this time (Ahmed).

Aleen who is a high school teacher for Deaf students stated that:

To be honest, no one asked us to complete our teaching services because all administrators were busy helping the majority [students without disabilities]. In addition, there is nothing for Deaf students. We [teachers of Deaf students] duplicated the first semester grades [the grades of semester 1, 2020 Χ 2] of our students (Aleen).

All seventeen responses affirm how Deaf students were directly excluded from any formal education service ever since schools were suspended by the Saudi government as a response to this pandemic.

### 3.4 Reasons for this full exclusion

All participants were asked about the reasons for excluding Deaf students from educational services during the suspension of schools resulting from the COVID-19 pandemic. Many of them (seventeen from eighteen) agreed that Deaf students were forgotten in this crisis and in many cases these students are treated as second-class citizens or as a secondary concern.

Fatima, who is a primary school teacher for Deaf students stated that:

This pandemic shifted the attention and efforts of the Ministry of Education to students without disabilities as they are the majority. Deaf students were forgotten in this case (Fatima).

Additionally, Ryan, who is a middle–school teacher for Deaf students noted that:

Deaf students in many cases are considered second-class citizens, especially in the last few years. And for sure, in this crisis, they were forgotten. All the focus tends to be on ‘normal’ students [students without disabilities] and Deaf students are not a priority (Ryan).

Omer who is a high school teacher for Deaf students confirmed that:

Indeed, our students and all Deaf students faced indifference from the Ministry of Education during this pandemic. Honestly, not from the Ministry only but from many sides … from schools, parents, press and us as teachers as well. These students need group efforts and cooperation, but they are facing carelessness from many directions (Omer).

The above reason was the major barrier and all the teachers argued that if the Ministry of Education moves towards providing alternative educational methods for Deaf students during this pandemic, it will help overcome every obstacle mentioned. However, to investigate other possible reasons, the participants were asked to discuss other possible causes for this exclusion.

Another reason for the exclusion of Deaf students during the suspension of schools is difficulties related to distance education. For instance, all participants considered the availability of internet services as the most important barrier to providing remote educational services to Deaf students. Jamal, who is a primary school teacher for Deaf students claimed that:

The best way to continue the provisions of educational services for these students is through the IEN TV Channels. It is cheaper and easy to access because many Deaf students come from low-income families where offering internet service is very difficult (Jamal).

Another example from Abeer, who is a middle–school teacher for Deaf students stated:

Distance education for Deaf students without the support of the Ministry of Education is inefficient because the majority of Deaf students cannot get good internet services for this type of educational method by themselves or by their families. It is costly (Abeer).

Additionally, Aamaal, who is a high school teacher for Deaf student noted that:

Many of these students come from poor families and/or rural areas where the availability of the internet for online learning is difficult without cooperation between the Ministry of Education and other governmental bodies such as the Ministry of Communications and Information Technology to offer such required services (Aamaal).

Finally, all participants in this study agreed that the absence or lack of electronic educational content for Deaf students in Saudi Arabia constitutes a big challenge for providing online learning for these students prior to and during the suspension of schools.

There is difficulty in finding local online content for Deaf students. Before this pandemic, I faced difficulties in offering electronic tools or educational contents for my students when I tried to use the internet to support my teaching process (Slma).

Another example, Faisal, who is a middle–school teacher for Deaf students claimed that:

Prior to this problem [COVID-19 pandemic], there were no online educational contents for Deaf students that were organised by specialists from Saudi Arabia because the sign language needs to come from a Saudi context. There are only a few YouTube videos that are done by unqualified people (Faisal).

From the above quotes, four main reasons for excluding Saudi Deaf students became apparent. These reasons are: shifting all the attention to the hearing students during this crisis, treating Deaf students as second–class citizens, indifference from many sides and the absence of alternative distance educational methods for these students. The next section discusses these findings.

## 4 Discussion

This current study provides a closer view of the status of Deaf students in Saudi Arabia during the suspension of schools resulting from the COVID-19 outbreak. Specifically, it covers the educational situation after this suspension from observing and analysing some of the provided alternative methods from the Ministry of Education and gathered data from the participants of this study. Thus, the findings include teachers' responses to students' full exclusion and some reasons for this exclusion.

In addition to the exclusion of all Deaf students in Saudi Arabia from general schools and excluding them in isolated classrooms or ‘special’ schools (Madhesh, 2019) 176), they were fully excluded from any alternative educational services during the suspension of schools. The academic year was over for Deaf students in Saudi Arabia when the suspension was implemented and they were given the same grades as the previous semester. In emergencies, such as the COVID-19 pandemic that happened with no notice or preparation, many vulnerable groups are ignored or forgotten. Indeed, disasters, crises and emergencies tend to increase systematic, or systemic, exclusion and/or discrimination against people with disabilities (Kelman and Stough 2015; Wisner et al. 2012). According to Qi and Hu (2020, 852), ‘the needs of persons with disabilities go unnoticed and are not met in emergencies and public health crisis’. Unfortunately, many countries do not have inclusive emergency plans for people with disabilities during crisis times (Pyke 2019). In Saudi Arabia, for example, there are some policies and laws that elucidate the rights of people with disabilities, such as the Disability Law, The Authority for the Care of People with Disabilities and Basic Regulations for Persons with Disabilities Rehabilitation Programs (Unified National Platform 2020). But there is no mention or text about their rights during emergencies and times of crisis. Clearly, ‘all too often, disabled people [people with disabilities] are left behind in emergencies, and this is a risk in the ongoing COVID-19 pandemic’ (Kuper et al., 2020, 3).

The full exclusion of Deaf students during this suspension was met with a lack of reaction by many teachers according to the participants' responses. Only one unsuccessful attempt was made by one participant to provide some help. This situation led to the speculation as to whether teachers of Deaf students neglected their responsibilities or whether they were unable to provide any help. According to the first viewpoint, the absence of accountability that results from the difficulty of accurately measuring educational achievement for many students with disability including Deaf students (Wolf and Hassel 2001) may lead to carelessness among some teachers of these students. However, the findings confirm that the inability of these teachers in providing alternative educational services for Deaf students during this pandemic stems from a lack of support from governmental bodies. According to a current study conducted by Fernández-Batanero et al. (2018), teachers of students with disabilities have low skill levels in understanding Information and Communication Technologies (ICT) and using this technology with their students. In the case of this current study, although this issue was mentioned, other reasons were reported by the participants.

For instance, all participants agreed that the exclusion of Deaf students from alternative educational methods was mostly because these students are treated differently by the Ministry of Education. They argued that Deaf students are treated as second-class citizens or as a secondary concern in this crisis and before it as well. Slee (2019) argues that many educational systems consider students with disabilities as additional students that require more funding. Additionally, these students are treated and seen as a ‘surplus population’ (Best 2019, 91) that are given the surplus of time, resources and attention. In most cases, the educational systems are designed for students without disabilities (Tijsseling 2015). Indeed, ‘they [students with disabilities] inhabit the margins of schooling, looking on at the education main game’ (Slee 2018, 4).

Moreover, difficulties facing distance education constituted another reason for the full exclusion of Deaf students during the suspension of schools. For example, the lack of access to internet services for many Deaf students leads to the futility of online learning provisions. High-speed internet service is a fundamental cornerstone for successful distance education (Simonson et al. 2019). According to Mthethwa-Kunene and Maphosa (2020), the poor infrastructure for proper internet services is a fundamental disruption for providing an adequate distance education service. Additionally, most Deaf students in Saudi Arabia come from poor families and/or live in rural areas (Bahatheg, 2015), which increases their deprivation of access to online educational services. Mohmedsalim et al., (2017) argue that students who live in rural regions find the required technologies for distance education including internet services are expensive and difficult to obtain.

Another reason for the full exclusion of Deaf students during this pandemic is the absence or lack of electronic educational contents for these students. McKeown and McKeown (2019) argue that Deaf students face three levels of barriers when accessing online content. These barriers include learning management systems (LMSs) that may constitute a difficulty for Deaf students to access some online learning websites or platforms. The second level of barriers is accessing the electronic materials for the course itself. Finally, the third level of barriers is accessing the communication tools for electronic educational content where most do not meet the needs of Deaf students.

## 5 Conclusion

Deaf students in Saudi Arabia were fully excluded from alternative education methods during the suspension of schools in the ongoing COVID-19 pandemic. Additionally, there were no efficient reactions from their teachers to resist this exclusion or to offset the absence of educational provisions for these students. These poor reactions may be a result of shortened efforts or the inability of these teachers to provide proper assistance. Although this current study slightly covers this point, it needs a more and deeper investigation.

This full exclusion is attributed to various reasons, such as the absence of an inclusive emergency plan, treating Deaf students differently as second-class citizens and/or surplus students, difficulties facing online learning and a lack of suitable electronic contents that meet the needs of Deaf students.

In short, the Ministry of Education in Saudi Arabia needs to rethink its related policies for people with disabilities, including Deaf students to create new inclusive policies or add new contents to current policies that recognise the needs of these individuals during emergency times. Additionally, it needs to review and update telecommunications services to cover all areas and offer internet services to all students with disabilities to avoid the huge effects of education suspension during crises such as the ongoing COVID-19 pandemic. Moreover, educational organisations are required to improve their online learning platforms to make them friendly and accessible for all students, including students with disabilities.

## Declarations

### Author contribution statement

Abdullah Madhesh: Conceived and designed the experiments; Performed the experiments; Analyzed and interpreted the data; Contributed reagents, materials, analysis tools or data; Wrote the paper.

### Funding statement

This research did not receive any specific grant from funding agencies in the public, commercial, or not-for-profit sectors.

### Data availability statement

The data that has been used is confidential.

### Declaration of interests statement

The authors declare no conflict of interest.

### Additional information

No additional information is available for this paper.
